# Mycobiome and Cancer: What Is the Evidence?

**DOI:** 10.3390/cancers13133149

**Published:** 2021-06-24

**Authors:** Natalia Vallianou, Dimitris Kounatidis, Gerasimos Socrates Christodoulatos, Fotis Panagopoulos, Irene Karampela, Maria Dalamaga

**Affiliations:** 1 First Department of Internal Medicine, Evangelismos General Hospital, 45-47 Ipsilantou Str., 10676 Athens, Greece; dimitriskounatidis82@outlook.com (D.K.); f_1992@hotmail.com (F.P.); 2 Department of Biological Chemistry, Medical School, National and Kapodistrian University of Athens, 75 Mikras Asias, Goudi, 11527 Athens, Greece; gerchristod82@hotmail.com; 3 Second Department of Critical Care, Attikon General University Hospital, Medical School, National and Kapodistrian University of Athens, 1 Rimini St, Haidari, 12462 Athens, Greece; eikaras1@gmail.com

**Keywords:** cancer, colorectal cancer, fungi, head and neck cancer, microbiome, mycobiome, pancreatic cancer

## Abstract

**Simple Summary:**

Although comprising a much smaller proportion of the human microbiome, the fungal community has gained much more attention lately due to its multiple and yet undiscovered interactions with the human bacteriome and the host. Head and neck cancer carcinoma, colorectal carcinoma, and pancreatic ductal adenocarcinoma have been associated with dissimilarities in the composition of the mycobiome between cases with cancer and non-cancer subjects. In particular, an abundance of *Malassezia* has been associated with the onset and progression of colorectal carcinoma and pancreatic adenocarcinoma, while the genera *Schizophyllum*, a member of the oral mycobiome, is suggested to exhibit anti-cancer potential. The use of multi-omics will further assist in establishing whether alterations in the human mycobiome are causal or a consequence of specific types of cancers.

**Abstract:**

Background: To date, most researchhas focused on the bacterial composition of the human microbiota. In this review, we synopsize recent data on the human mycobiome and cancer, highlighting specific cancer types based on current available evidence, presenting interesting perspectives and limitations of studies and laboratory methodologies. Recent findings: Head and neck cancer carcinoma (HNCC), colorectal carcinoma (CRC) and pancreatic ductal adenocarcinoma (PDA) have been associated with dissimilarities in the composition of mycobiota between cancer cases and non-cancer participants. Overall, fungal dysbiosis with decreased fungal richness and diversity was common in cancer patients; however, a specific mycobiotic signature in HNSCC or CRC has not emerged. Different strains of *Candida albicans* have been identified among cases with HNCC, whilst *Lichtheimia corymbifera*, a member of the *Mucoraceae* family, has been shown to predominate among patients with oral tongue cancer. Virulence factors of *Candida* spp. include the formation of biofilm and filamentation, and the secretion of toxins and metabolites. CRC patients present a dysregulated ratio of *Basidiomycota/Ascomycota.* Abundance of *Malassezia* has been linked to the occurrence and progression of CRC and PDA, particularly in animal models of PDA. Interestingly, *Schizophyllum*, a component of the oral mycobiome, may exhibit anti-cancer potential. Conclusion: The human mycobiome, per se, along with its interactions with the human bacteriome and the host, may be implicated in the promotion and progression of carcinogenesis. Fungi may be used as diagnostic and prognostic/predictive tools or treatment targets for cancer in the coming years. More large-scale, prospective, multicentric and longitudinal studies with an integrative multi-omics methodology are required to examine the precise contribution of the mycobiome in the etiopathogenesis of cancer, and to delineate whether changes that occur in the mycobiome are causal or consequent of cancer.

## 1. Introduction

**Figure 1:**
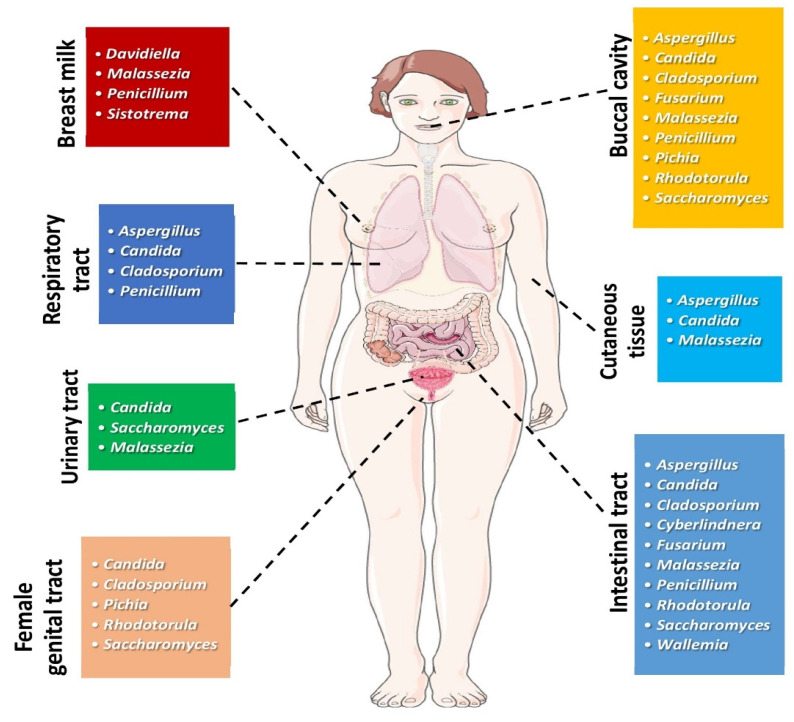
Most common genera of fungi found in different human body sites under physiologic conditions. (All images originate from the free medical website http://smart.servier.com/ (accessed on 25 May 2021) by Servier licensed under a Creative Commons Attribution 3.0 Unported License).

Fungi have recently been estimated to consist of up to 3.8 million species; thus, they represent a taxonomic and functional diversity of life forms, being implicated in complex and yet unknown interactions with other living microorganisms [1]. Fungi are microeukaryotes and constitute a smaller part of the human microbiome in comparison to bacteria, forming the so-called “human mycobiome” [2,3,4]. Fungal communities can be found in different anatomic sites of the human body, as depicted in Figure 1.

The number of human microbiota has been determined to be 10^14^, about 10 times greater than the number of human cells. Also, the quantity of microbial genes is about 100 times more than the corresponding quantity of human genes. The human mycobiome accounts for approximately 0.001% to 0.1% of the microbial community in the gut [5,6]. Over the last years, fungi have been the subject of intense investigation, with a particular focus on their contribution to human disorders, especially among immune-compromised patients [7]. However, as most fungi are not easily cultured, even in specific cultural media, their study has been limited until today, due mainly to the unavailability of methods used for their detection. Nevertheless, genomic methodology in fungi research may broaden our knowledge in their contribution to health and disease [2,3,4]. High-throughput sequencing (HTS) analysis of fungi is reshaping the area of the fungal community [1].

In the gut, bacteria outnumber fungi, but we cannot overlook the fact that fungal taxa may merely be determined with modern sophisticated, non-culture-based methods. Despite the fact that the gut mycobiome is less analyzed than the bacteriome, it seems likely that fungi are primarily spread intra-uterinally to the fetus [8]. Recent studies have suggested that fungi are found in the gut microflora of young children via transmission from their mother, siblings and environmental exposure; nevertheless, diet may be the most significant factor [9]. Dietary intake plays a key role, as fungi colonize the gut by food digestion. Fungi which colonize the intestines via dietary intake could be part of the gut flora or be rejected [3]. Despite the paucity of studies, the importance of dietary intake in the content of the intestinal mycobiome is confirmed by the fact that vegetarians present dissimilarities in the mycobiotic composition in comparison to those following a Western-style nutrition [3,10]. The interplay of intestinal mycobiome with bacteriome and virome is a hot topic of research, especially in the field of mycobiome-associated diseases.

Current scientific evidence has supported the contribution of the intestinal mycobiome in affecting immune response, with an impact on regional and systemic disease [11]. Notably, a considerable number of pathogenic fungi are “pathobionts”, i.e., residents in the organism that are not implicated in the pathogenesis of any disorders under physiologic circumstances but that may exhibit pathogenetic properties. Following this trend, *Candida albicans*, which belongs to the physiologic intestinal ecosystem, is the etiologic agent of systemic candidiasis in immune-compromised subjects [12]. The transformation of non-pathogenic fungi under physiologic circumstances to pathogenic fungi under unspecified conditions is a subject of intense research. Indeed, fungal diseases constitute a considerable part of the totality of the infectious disease range. A substantial part of infections includes fungal infections in immune-compromised subjects with an approximate death rate of 35% to 45% [12]. However, there is currently growing interest in the associations between the human mycobiome and its potential role in human carcinogenesis. In this comprehensive review, we present a synopsis of recent data on the human mycobiome and cancer, focusing on specific cancer types based on current available scientific evidence, giving an emphasis on the interplay among the human mycobiome, microbiome and the host influencing carcinogenesis.

## 2. Mycobiome and Head and Neck Cancer

Head and neck cancer is the 6th most frequent malignancy globally, with oral cancer (OC) and oropharyngeal carcinoma (OPC) being the most common types. Approximately half of the cases of OC and OPC have topical or remote metastases at diagnosis, thus resulting in a 50% death rate [13,14]. The risk factors of head and neck squamous cell carcinoma (HNSCC) have not been elucidated until today. Main etiologic factors of HNSCC include human papilloma virus (HPV), tobacco, genetic predisposition, UV radiation, alcohol consumption, occupational exposure to wood and coal dust, asbestos, formaldehyde, and nutrition poor in vegetables and fruits [14,15].

The role of the mycobiome in OC and OPC has not been thoroughly investigated. *Candida* spp. are the most commonly encountered fungi in the oral mycobiome among healthy adults, followed by *Cladosporium*, *Aureobasidium*, *Saccharomycetales*, *Aspergillus*, *Fusarium* and *Cryptococcus*. In particular, *Candida*, *Aspergillus*, *Fusarium* and *Cryptococcus* represent the leading genera, and are considered pathogenic fungi in humans [16]. Nevertheless, there is a paucity of data regarding the oral fungal community amid patients with cancer. Shelburne et al. have studied host whole exome sequencing as well as genetic analysis of infectious agents, and have determined the oral and fecal microbiome and mycobiome in a patient with leukemia. They concluded that bacterial dysbiosis in the oral cavity could provide a permissive milieu for the subsequent emergence of invasive mucormycosis [17]. Furthermore, recent studies have highlighted the importance of the interplay between bacterial and fungal communities, i.e., inter-kingdom interplay. These studies have pointed out that the bacteriome or the mycobiome could contribute to the pathogenesis of various diseases, but their interaction may also have an important impact [17]. In order to examine the interaction between oropharyngeal bacteriome and the mycobiome, Mukheerjie et al. have focused on random forest modeling of an oral mycobiome and bacteriome [18]. Amid the predominant parameters, this model has detected ten genera of bacteria, such as *Rothia*, *Eikenella*, *Streptococcus*, *Porphyromonas*, *Aggregatibacter*, *Fusobacterium*, *Prevotella*, *Actinomyces*, *Campylobacter*, *Capnocytophaga*, and only one genus of fungi, *Emericella,* Afterwards, they performed inter-and intra-kingdom association analyses with taxa belonging to bacteria and fungi in the microbiota of 39 oral tongue cancer and non-tumor samples. They have demonstrated that *Bacteroidetes* showed positive intra-kingdom associations with *Fusobacteria* and *Spirochaetes* in cancer samples. In parallel, there was a negative relationship between *Zygomycota* and *Ascomycota*, whilst the association between *Glomeromycota* and *Ascomycota* was reduced in cancer samples. In addition, *Zygomycota* had a positive inter-kingdom relationship with *Fusobacteria* and *Bacteroidetes*, and a negative relationship with *Actinobacteria*. Fungal species such as *Lichtheimia* presented a positive association with *Campylobacter*, *Porphyromonas* and *Fusobacterium*, and a negative one with *Actinomyces. Lichtheimia corymbifera*, a member of the *Mucoraceae* family in the *Zygomycota* phylum, was found to be positively related to eleven bacteria and negatively to thirty-nine bacteria, among which was *Lactobacillus* spp. These findings shed light on the specific inter- and intra-kingdom relationship that may take place in the bacterial and fungal communities in the context of oral tongue cancer [18].

Given the complexity of carcinogenesis, we may hypothesize that many genomic and epigenomic loci exhibit alterations in head and neck malignant neoplasms, confirming the multi-hit process of malignancy. Mukheerjie et al. have documented a similar multi-hit process of bacteriome and mycobiome in the etiopathogenesis of oral carcinogenesis. Alterations in the oral microbiome and mycobiome may account for cancerous effects of metabolites secreted by these microorganisms. In this context, acetaldehyde, which is produced by alcohol metabolism, was suggested to be linked to OC related to chronic alcohol intake. Due to chronic alcohol consumption and abundance of bacteria which synthesize acetaldehyde, including *Rothia*, *Streptococcus* and *Prevotella*, higher oral acetaldehyde may be implicated in oral tumorigenesis [18]. Cancer and non-cancer groups presented differences in fungal abundance. Some fungi could exhibit an oncogenic potential, as shown with *Candida albicans*, which may participate in the synthesis of salivary acetaldehyde in subjects with ethanol-associated OC [19,20,21,22,23]. More research is needed to also explore the carcinogenic properties of the fungi *Lichtheimia corymbifera*. It is noteworthy that correlation analyses have also documented a negative association between *Lichtheimia corymbifera* and *Lactobacillus* spp., that may be associated with alterations in the regional intestinal milieu that enhances the overgrowth of particular taxa. *Lactobacillus* spp. are considered favorable bacteria that modulate the development of bacterial and fungal communities [3,23]. A decrease of *Lactobacillus* spp. could cause perturbations in the microbial microflora of patients suffering from oral tongue cancer. This imbalance in the microbial ecosystem may interfere with factors, such as pH, and/or micronutrients, which predispose to microbial dysbiosis [23].

Very recently, Shay et al. have studied the bacterial and fungal communities as well as their interplay in the oral wash of forty-six subjects with HNSCC and a similar number of non-HNSCC individuals [24]. Oral wash samples were collected for microbiome studies. They have detected three phyla of fungi and eleven phyla of bacteria. *Ascomycota* from the fungal community (72%) and *Firmicutes* from the bacterial community (39%) were the predominant microorganisms. Notably, strains of *Candida albicans* and *Rothia mucilaginosa* presented differences in abundance, whereas *Schizophyllum commune* was diminished in the oral wash from subjects suffering from HNSCC in comparison to non-HNSCC individuals. Collectively, these findings highlight the existence of differences in abundance of bacterial and fungal communities as well as the microbiome–mycobiome interactions in the oral wash of subjects with HNSCC, in comparison to non-HNSCC participants. In particular, specific strains of *Candida albicans* were over-presented or under-presented in the oral wash samples from subjects with malignancies, when compared to samples from non-HNSCC participants. *Candida dubliniensis*, *Schizophyllum commune* and a fungus from the class of *Agaricomycetes* were over-represented in controls in comparison to cancer patients. On the contrary, one fungal strain of *Neoascochyta exitialis* was under-represented in the oral wash from subjects with HNSCC, in comparison to controls. *Candida* was the predominant fungal genus in the oral fungal microflora of both patients with HNSCC and non-HNSCC participants [24]. This finding has been observed across many studies examining the oral mycobiome among patients and controls [25,26,27].

Oral candidiasis has been related to the development of malignancies, such as head and neck malignancies [25,26,27]. Perera et al. have detected an overgrowth of *Candida albicans* in the oral squamous cell malignant tissue in comparison to benign tissue (intra-oral fibro-epithelial polyps) [26]. Vesty et al. have noted an enrichment of *Candida albicans* in the saliva of subjects with HNSCC patients, which correlated with an increase in the inflammatory cytokines interleukin (ΙL)-1β and IL-8 [27]. The latter observation is suggestive of the potential contribution of *Candida albicans* to the promotion of inflammation and carcinogenesis through hyper-methylation of various tumor suppressor genes [28,29]. In addition, *Candida albicans* is known to produce biofilms, which form a resistant shield that protects the fungal community from external factors, and are related to improper immune elimination by the host. Fungal filamentation is also a known *Candida* virulence factor, which also damages host tissues and triggers host inflammatory response [30]. Nevertheless, abundance of *C. albicans* in both healthy participants and patients does not provide enough evidence that this organism may be implicated in HNSCC carcinogenesis [30,31,32]. It is plausible that the study by Shay et al. identified both pathogenic and non-pathogenic *C. albicans* strains. Further research is necessary to characterize those *C. albicans* strains that are related to HNSCC [24]. This characterization could increase the specificity of a microbiome-based oral wash screening tool for HNSCC. Apart from the differential species of *C. albicans*, a second fungi, *Schizophyllum commune*, was in abundance in the oral wash of healthy controls. The genera *Schizophyllum* is a member of the phylum *Basidiomycota,* and has been known as a member of the oral mycobiome [33,34,35]. *Schizophyllum commune* is suggested to produce the polysaccharide compound schizophylan [36]. Schizophylan has anti-cancerous properties in vitro and has shown promise in the treatment of cancer patients, including HNSCC, in studies conducted in Japan in the 1980s [35,36,37,38,39]. The abundance of *Schizophyllum commune* among controls supports its role as a potential anticancer agent. Table 1 depicts the main studies associating the mycobiome with neoplastic diseases in animal models and in humans.

Overall, while some *C. albicans* strains are involved in the etiopathogenesis of HNSCC, other strains are not participating. Moreover, *Schizophyllum commune* seems to be protective against HNSCC. It remains to be elucidated whether it is just the specific strains or the inter-kingdom interplay with large-scale, longitudinal, multi-omics studies combining metagenomics and metabolomics.

## 3. Mycobiome and Colorectal Cancer (CRC)

CRC is the third most frequent causal factor of cancer mortality in both genders in the United States, with an estimated incidence of approximately one million patients annually, worldwide. In addition, a considerable number of patients with CRC are younger and present with advanced stage of cancer [45,46,47]. CRC morbidity and mortality may be diminished by appropriate screening and surveillance [48,49].

Notably, more than 50% of cancer cases and deaths are attributed to modifiable predisposing factors, including Western-type nutrition based on less intake in vegetables and fruit, higher intake of alcohol, lack of somatic exercise, smoking, and overweight/obesity. Moreover, the gut bacteriome, particularly *Enterococcus faecalis*, *Escherichia coli*, enterotoxigenic *Bacteroides fragilis*, *Streptococcus bovis* and *Streptococcus gallolyticus*, has been involved in colorectal oncogenesis [50]. Alterations in gut microbiota may interfere with environmental parameters, affecting the risk for CRC. Environmental predisposing factors may change the composition and properties of the gut microbiota, in conjunction with the immunometabolic networks that play an important role in colorectal carcinogenesis [51].

### 3.1. The Role of Fungal Dysbiosis in CRC

Besides bacteria inhabiting the gastrointestinal (GI) tract, fungal phyla, such as *Basidiomycota*, *Glomeromycota* and *Ascomycota*, reside in high numbers in the digestive tract [47]. The most commonly found fungal genera inhabiting the physiologic GI are *Candida*, *Saccharomyces*, *and Cladosporium* [47]. Trojanowska et al. demonstrated that the intestinal tract is also inhabited by members of the oral mycobiome, as they have identified the same *Candida albicans* strain in the oral cavity and gut of subjects with inflammatory bowel disease (IBD) [52]. Unfortunately, there is a paucity of data regarding gut fungal commensals in cancer. Dysbiosis is well-known among patients suffering from IBD, who present higher odds of CRC occurrence [17]. It is noteworthy that decreased richness and diversity have also been reported in the bacterial community as well as the fungal microbiome [17,47]. For example, *Cystofilobasidiaceae*, *Dioszegia* genus and *Candida glabrata* were detected in abundance in the gut of subjects suffering from Crohn’s disease, when compared to healthy individuals [53]. Luan et al. have focused upon comparing the mycobiota composition in adenomas and their normal adjacent colon tissues. They have documented an increased number of *Phoma* and *Candida* genera as well as *Candida tropicalis* in adenomas [40]. These fungi may act as pathobionts, being implicated in tumor onset and progression.

Patients with CRC have been documented to present an increased ratio of *Basidiomycota/Ascomycota* [41,48]. Patients with colitis-associated CRC have also shown a similar ratio [47]. Coker et al. have detected 14 fungal biomarkers with a differential abundance in 184 CRC patients in comparison to 204 healthy participants [43]. Moreover, an abundance of *Malassezia* has been found among CRC patients by fecal shotgun metagenomic sequencing in conjunction with *Moniliophthtora*, *Rhodotorula*, *Acremonium*, *Thielaviopsis* and *Pisolithus*, whilst an increased number of *Basidiomycota* have been suggested to be related to more advanced stages of the disease [42,43,54].

**Figure 2:**
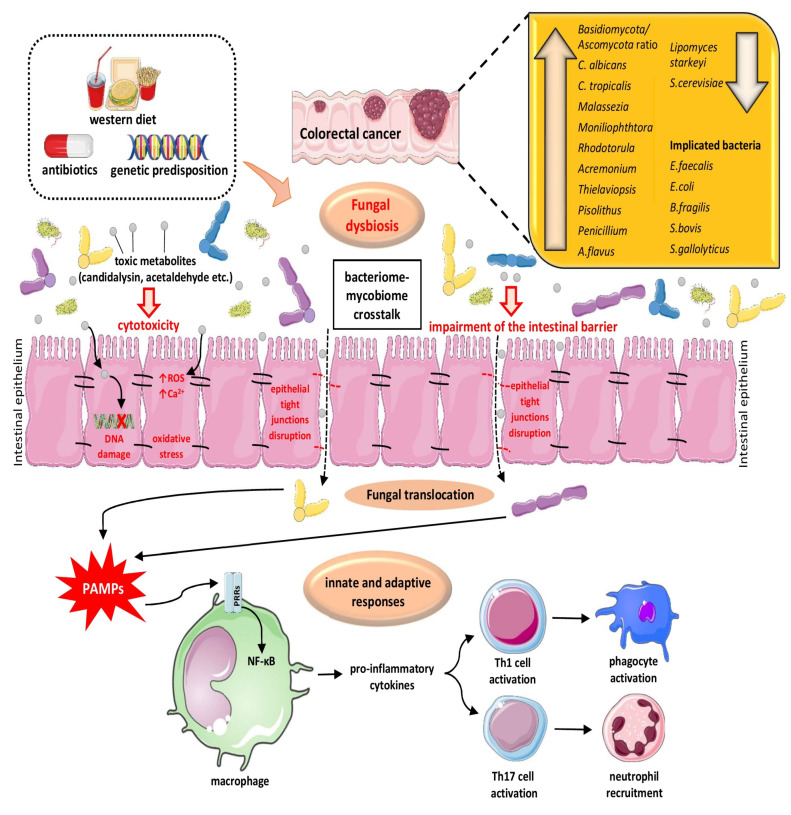
Fungal dysbiosis is the result of a multi-factorial process involving various environmental parameters, including Western-type nutrition and chronic administration of antibiotics as well as genetic predisposition, all resulting in impairment of the intestinal barrier and cellular tight junctions. This dysfunction induces opportunistic fungi translocation with consequences in the host innate and adaptive immune system. In macrophages, the interaction of fungal cell wall elements (PAMPs) by PRRs (e.g., CLRs) induces the secretion of pro-inflammatory cytokines, which leads to the Th1 and Th17 cells activation, provoking phagocyte stimulation and neutrophil chemotaxis. Furthermore, harmful metabolites produced by pathogenic fungi, such as acetaldehyde from *Candida albicans* and *Candida tropicalis*, and candidalysin from *Candida albicans*, may induce cytotoxicity and DNA damage, by provoking oxidative stress, via increased ROS production and the enhancement in intracellular Ca^++^ levels. The interaction between the bacteriome and the mycobiome, i.e., the inter-kingdom interplay, is equally or even more important in the process of carcinogenesis in CRC. Abbreviations: Ca^++^: calcium cations; CLRs: C-type lectin receptors; CRC: colorectal cancer; NF-κB: nuclear factor-kappa B; PAMPs: pathogen-associated molecular patterns; PRRs: pattern recognition receptors; ROS: reactive oxygen species; Th cell: T helper cell (All images are originated from the free medical website http://smart.servier.com/ (accessed on 25 May 2021) by Servier licensed under a Creative Commons Attribution 3.0 Unported License).

Notably, a higher ratio of *Basidiomycota/Ascomycota*, an enhancement in *C. albicans* and *C. tropicalis* and a reduction in *Saccharomyces cerevisiae* were documented in individuals with IBD. It is noteworthy that *C. albicans* may produce a cytolytic toxic peptide called candidalysin, which is known to promote disruption of the epithelial barrier function, thus mediating dysbiosis. In addition, *C. albicans* and *C. tropicalis* may produce acetaldehyde to carcinogenic levels. Acetaldehyde is suggested to increase intracellular reactive oxygen species (ROS) and Ca^++^ concentrations, thereby causing mitochondrial dysfunction, leading to cytoxicity as well as the disruption of epithelial tight junctions [47]. Figure 2 depicts various mechanisms by which fungal dysbiosis may participate in the etiopathogenesis of CRC. Overall, mycobiota dysbiosis is suggested to be a triggering factor of CRC among subjects with IBD through chronic inflammation and secretion of toxic metabolites, which may cause DNA damage.

### 3.2. The Interplay of Gut Microbiome and Mycobiome in Colon Physiology/Pathology and CRC Pathogenesis

Recent data have shown that the interplay between intestinal fungal and bacterial communities may affect the intestinal microbiome homeostatic balance maintaining overall intestinal health and protecting from gastrointestinal disorders. Prolonged antifungal treatment resulted in an exacerbation of colitis and alteration of the gut bacteriome in a mouse model treated with dextran sulfate sodium (DSS), which provokes colitis [55]. In a murine model, intake of the pathobiont fungus *Mucor circinelloides* resulted in a reduction of the beneficial *Akkermansia* and an augmentation of *Bacteroides* genus [56]. *Candida albicans* restored bacterial variability and influenced the bacterial colonization of the gut after broad-spectrum antibiotic treatment, such as the increase in *Bacteroides* species and the pathogen *Enterococcus faecalis*, and the reduction in *Lactobacillus* spp. [57,58].

Intestinal fungi and bacteria interact through a variety of ways, including the secretion of metabolites and toxins, the development of biofilms, and physical attachment, thus influencing host immune responses. Based on in vitro and in vivo research data on bacterial and fungal interplay, it was shown that synergistic associations generally enhance pathogenicity whilst antagonistic relations limit bacterial or fungal virulence [59].

Some examples of synergistic actions between bacteria and fungi that may enhance colitis are the following: (1) the requirement of *Enterobacteriaceae* that aggravate DSS-induced colitis mediated by *Candida albicans* [60]; (2) the enhancement of the strict anaerobe *Clostridium difficile* by *Candida albicans* because of the oxygen decrease in the proximity of the yeast [61]; (3) the survival at decreased pH of *Helicobacter pylori* in the vacuoles of *Candida albicans* [62].

Some examples of antagonistic actions between bacteria and fungi with beneficial or neutral actions in gut health include: (1) the antifungal activity of *Serratia marcescens*, *Salmonella typhimurium* and *Acinetobacter baumannii* on eliminating the hyphal and yeast forms of *Candida albicans* or limiting the formation of biofilm and infection [59]; (2) the restriction of hyphal growth of *Candida albicans* by *Clostridium difficile* via the production of p-cresol [63].

Biofilms represent aggregations of microorganisms that are embedded in an extracellular polymeric matrix sticking to biological or non-living surfaces. The development of biofilm is enhanced by the collaboration of *C. albicans* and bacterial microorganisms including, among others, *E. coli*, *E. faecalis*, *Streptococcus* spp., *Staphylococcus aureus*, and *Staphylococcus epidermidis,* while other bacteria including *K. pneumoniae* and *P. aerugivalis* limit the synthesis of biofilm [47]. The intestinal mucosal biofilm may be an inducing factor in colorectal carcinogenesis [64]. Biofilms from subjects with CRC or healthy individuals submitted to colonoscopy triggered tumorigenesis in mouse models of CRC through promotion of chronic inflammation, evasion from the host immune system and disruption of epithelial integrity [64]. A close physical contact between *Candida tropicalis* and *E. coli* facilitated by *Serratia marcescens* was observed by electron microscopy in subjects with Crohn’s disease, an IBD which predisposes patients to small bowel and colon cancer [65]. This fungal–bacterial interaction created a robust biofilm which triggered sustained intestinal inflammation. Based upon these findings, it can be inferred that similar biofilms create the perfect persistent inflammatory milieu for the promotion of colon carcinogenesis.

Another important aspect in colorectal carcinogenesis is that the majority of pathogenic bacteria associated with CRC resides also in the oral cavity. Interestingly, a plethora of studies have shown that the oral bacterial pathogens *Fusobacterium nucleatum* and *Porphyromonas gingivalis* could contribute to the initiation and progression of CRC and pancreatic cancer via chronic inflammation, inhibition of host immunity and the secretion of tumorigenic substances [66,67,68]. A microbiota and metabolomics profiling on feces from CRC patients and matched controls has shown that metabolites were linked to CRC via their association with *Fusobacterium* and *Porphyromonas* [69]. Interestingly, *Fusobacterium nucleatum* has been shown to form coaggregations with both the hyphal and yeast forms of *Candida albicans* through the involvement of genetic and structural cellular components [70]. This coaggregation may contribute to facilitate their synergistic colonization in the oral cavity and the gastrointestinal tract, as well as to enhance pathogenesis and chronic inflammation. However, more mechanistic and clinical studies are needed to decipher the implications of this coaggregation in colorectal carcinogenesis.

Finally, a growing body of evidence has suggested that there is a significant interplay between gut microbiota and the host at the intestinal stem cell niche level, where microbiota may affect directly or indirectly the proliferation, differentiation and reprogramming of the intestinal stem cells and their transformation to cancer stem cells, resulting in CRC initiation and progression through a plethora of mechanisms reviewed elsewhere [71]. The totality of studies has focused on the role of gut bacteria on the abnormal reprogramming of intestinal and cancer stem cells without examining the role of fungi in this interplay. More studies with the use of integrative system-based approaches (i.e., metagenomics) are required to decipher the interplay of gut bacterial and fungal communities with intestinal and cancer stem cells in the initiation and promotion of colorectal carcinogenesis.

## 4. Mycobiome and Pancreatic Cancer

Pancreatic cancer represents the 7th leading cause of cancer mortality in both genders. Its incidence varies, being from 4-fold to 5-fold greater in elevated income countries, with the most increased incidence observed in Europe, Northern America, and Australia/New Zealand [48]. Both death as well as incidence rates have presented a plateau or have to some extent augmented, probably due to the increasing prevalence of obesity, diabetes mellitus, and chronic alcohol intake. However, amelioration in the currently available screening tools may also contribute to the increasing diagnosing rates [72]. Demographic factors, including age, sex and ethnicity/race have been considered risk factors of pancreatic cancer, while tobacco smoking and alcohol consumption represent two established environmental risk factors. Moreover, diabetes mellitus and obesity, particularly in men, have been lately related to an increased risk for pancreatic cancer [73,74].

The mycobiome has not been clearly involved in the carcinogenesis of pancreatic ductal adenocarcinoma (PDA), until only recently. Aykut et al. have shown that fungi may migrate from the intestinal lumen to the pancreatic parenchyma [44]. Notably, PDA has been found to harbor a ~3000-fold increment in fungi in comparison to a physiologic pancreas in both animal models and human studies [44]. The content of the PDA mycobiome was different from that of physiologic intestinal and pancreatic tissues based on specific diversity indexes. In particular, the mycobiome infiltrating PDA tumors was rich in *Malassezia*, in both rodents and humans. Fungal ablation with the use of the anti-fungal agent amphotericin B has been found to be tumor-protective in slowly progressive as well as in models of invasive PDA, whereas repopulation with *Malassezia,* but not with *Candida*, *Saccharomyces*, or *Aspergillus,* has been documented to provoke carcinogenesis in mice. Aykut et al. have reported that the connection of mannose-binding lectin (MBL), which attaches fungal wall glycans to enable the activation of the complement cascade, was responsible for neoplastic promotion, while MBL or C3 deletion in the extra-tumoral compartment or C3aR knockdown in cancer cells had been tumor-protective, even in the presence of *Malassezia*. Moreover, re-programming of the mycobiome has not changed PDA promotion in MBL or C3 deficient rodents. It is noteworthy to mention that Aykut et al. have shown that pathogenic fungi, such as *Malassezia*, promote PDA via exploiting the complement cascade by means of MBL activation. Based on data regarding the microbiome and the mycobiome, the oncogenic Kras-induced inflammation may induce fungal dysbiosis, which results in cancer progression through the stimulation of the MBL-C3 pathway [44]. Of note, based on the interrelationship between the mycobiome and the microbiome, more elaboratively designed studies using HTS are needed to estimate this bilateral inter-kingdom interaction in PDA [75]. Nevertheless, this outstanding study suggests that the mycobiome could represent a novel therapeutic target for pancreatic cancer in the near future.

## 5. Limitations of Studies and Laboratory Methodologies

### 5.1. Limitations and Challenges in Studies

Overall, fungal dysbiosis with decreased fungal richness and diversity is a common theme in cancer patients; however, a specific mycobiotic signature in HNSCC or CRC has not been revealed based on studies depicted in Table 1. Generally, studies on human mycobiome and cancer are scarce and heterogeneous, while they have included only a small number of individuals with cancer (from 14–184 patients), as they are expensive and need modern and sophisticated equipment. Therefore, due the small sample size, the statistical power to detect any differences in mycobiota between cases and controls is low. An important shortcoming of all studies is their retrospective or cross-sectional design, which is not appropriate to draw any conclusion regarding mycobiota as causal factors in cancer. Despite difficulties in performing such studies, more prospective, multicentric, larger and longitudinal studies over a long time period (≥5–10 years), are required to shed light on the role of the composition of fungal or other microbiota in cancer etiopathogenesis. The discrepancy of results between studies may also be attributed to the different specimens used (stool or saliva specimens versus tissue biopsies), different laboratory techniques summarized in 5.2, different populations and ethnic groups, different pathologic subtypes and stages of cancer examined, high inter-and intra-individual variability of the mycobiome, lack of evaluation criteria, etc. It is important to mention that more significant mechanistic evidence about the interplay of microbiota and host can be achieved by studying tissue biopsy specimens, due to the adherence of microbial communities to the epithelia, particularly the intestinal epithelium.

In addition, identification to the fungi species level is difficult, whilst it may be inconclusive and not illuminating. For example, *C. albicans* may be implicated in the etiopathogenesis of HNSCC, but it is also a component of the oral microflora among healthy individuals. Therefore, identification of different strains of *C. albicans* may be mandatory in order to shed light upon slightly differential, but potentially pathogenic, strains within the same species [24,30,31,32].

Finally, based on the paucity of available data, formal meta-analyses examining the association of a mycobiome with cancer occurrence, while significant, is very challenging to undertake.

### 5.2. Limitations and Challenges in Sample Collection and Laboratory Methodologies

Regarding fungal identification, as fungi are ubiquitously spread, there are concerns regarding the sampling and processing methods in order to avoid contamination [76]. Fungal DNA extraction is performed by mechanical cell lysis; however, there are some difficulties including their recalcitrant chitinous cell walls and the interference of secondary metabolites with DNA extraction. Therefore, the DNA extraction methods should be suitable for fungi. For example, species particularly rich in polysaccharides, such as encapsulated yeasts, may require further special DNA extraction techniques [77,78].

There is a plethora of laboratory methods to detect live fungi or a genome of fungal communities including, among others culture, fluorescent microscopy, amplicon sequencing and whole-genome shotgun (WGS) metagenomics, or high-throughput sequencing (HTS) metabarcoding studies [1,59,79]. Overall, sequencing methodologies reveal fungal DNA in a specimen independent of fungal viability.

In analogy to 16S sequencing for bacteria, amplicon sequencing employs distinct primers for fungi to amplify the internal transcribed spacer (ITS) region or the 18S region of the nuclear ribosomal RNA (rRNA) gene locus, permitting the discrimination of fungal species [59]. Sequencing the ITS region of the nuclear rRNA operon is the gold standard in Sanger sequencing-based species detection and HTS-based methods [80,81]. ITS constitutes the main fungal taxonomic marker gene or molecular identifier or “DNA barcode” [1]. Because the whole ITS region is too long for sequencing, spanning 500–700 bps, the majority of HTS-sequencing studies has concentrated on either the ITS1 or the ITS2 subregion within 250–400 bps [1,82]. The choice of what sub-region to use is still a discussed point. The use of ITS2 subregion may offer a more ubiquitous primer location and reduced length variability, resulting in fewer taxonomic errors in comparison to ITS1 [79]. Nevertheless, conflicting results have been emerged in the comparisons between the sub-regions ITS1 or ITS2 for the analyses of fungal profiles [83]. The advantages of using the full ITS region instead of ITS1 or ITS2 with third-generation HTS platforms such as Oxford Nanopore or Pacific Biosciences include the higher taxonomic discrimination and the decreased amplification of non-living microorganisms; however, a shortcoming is the low performance with poor-quality samples due to the DNA deterioration, where full ITS sequencing may be impossible [1]. Overall, the disadvantages of the amplicon sequencing comprise the paucity of a complete annotated reference database, taking into account the complex taxonomic composition of the fungal community as well as the amplification errors inherent to PCR [59].

Despite the fact that amplicon sequencing is the most widely used methodology to spot genes, shotgun HTS allows the detection of various genes together, based on the sequencing of all isolated genetic material in a given specimen without the employment of targeted PCR quantification of the ITS or other gene regions [79]. Notably, in comparison to amplicon sequencing, omics methods may confer taxonomic composition more precisely, by circumventing biases related to PCR and primer selection [84,85]. Besides, omics presents the ability to improve standardization as well as comparison of different phyla for future studies [86]. Moreover, metagenomics offers the opportunity to identify fungal taxa in conjunction with prokaryotes, and this dual ability may explain their usefulness in exploring different microbiomes [87,88].

The combination of a HTS approach with barcoding has been termed “metabarcoding”, and consists of several laboratory processes needing bioinformatics and computational statistical analyses. Briefly, a typical fungal metabarcoding workflow presents the following major items: specimen preparation, extraction of DNA, DNA amplification, HTS-sequencing, processing of sequencing, quality control (demultiplexing, elimination of chimeric molecules and flanking genes, etc.) and sequence data analysis (clustering, operational taxonomic unit/OTU positioning and taxonomic annotation) [1]. Therefore, the selection of an adequate modus operandi for all processes in the metabarcoding workflow is of paramount importance. Inadequate methodology in metagenomic studies may result in erroneous biological assumptions. HTS significant biases may happen from additive systematic and random errors, which include, among others, biases in genetic material extraction, markers, primers, PCR, library preparation, sequencing, bioinformatics analysis, index-switching, low clustering, unequal sequencing depth, etc. [1,83]. Moreover, DNA metabarcoding for microbiota analyses presents significant shortcomings, including the variability of copy numbers of the targeted barcodes in microorganism genomes, the poor taxonomic discrimination at the species level for some microbiota, and errors in the taxonomic assignment of sequences based on the selected variable region [83].

WGS metagenomic studies in a plethora of human specimens have shown the reduced quantity of fungi (≤0.1% of total microbiota) in comparison to bacterial DNA. Fungal DNA has a large quantity of non-coding regions compared to bacterial DNA, which further complicates fungal metagenomics [89]. Furthermore, genomic fungal databases have been lacking until today, especially when compared to genomic bacterial databases [89,90]. Due to the low sequence quantity rates, it is hard to implement OTU studies based on metagenomic data.

Fungal metatranscriptomics is a more beneficial, emerging and analytical approach because of the lack of introns in the expressed genes and the better annotation. It is noteworthy to mention that RNA-based studies may help in the quantification of fungal taxa as well as their functionality in the least biased way [1]. Metaproteomics and metabolomics help to understand the properties and the interplay between intestinal microbiota (e.g., bacteria and fungi), by providing information of the role of bacterial and fungal proteins and metabolites (i.e., small and low molecular weight molecules, being the downstream products of gene and protein processes), respectively. In the framework of cancer microbiota, a variety of metabolites may originate from bacterial, fungal and host metabolisms, or cometabolic networks between the host and microbial communities. In order to circumvent the compositional nature of metabarcoding data in the metagenomics strategy, a multi system-based approach (integrative multi-omics) could broaden our knowledge of the function of the interplay of microbial communities and cancer stem cells in carcinogenesis [71]. Llyod-Price et al. used an innovative multi-omics network analysis of ten omics data (metagenomic, metabolomic, metatrascriptomic data, etc.) to highlight the pathogenetic mechanisms in IBD [91]. A similar approach could be useful in deciphering the pathogenetic mechanisms in cancer. Furthermore, using integrative analytical approaches of metagenomic and small noncoding RNA-sequencing data from stool samples, a human and microbial small RNA signature was detected, and could be used for diagnostic purposes in CRC [92].

Finally, prospective, larger and long-term research studies are required to examine if alterations in the mycobiome are causal or a consequence of cancer.

## 6. Perspectives

Studies on the mycobiome may present important preventive, diagnostic and prognostic as well as therapeutic implications in cancer.

### 6.1. Preventive and Therapeutic Implications

Fungal dysbiosis may be reversed through nutrition, administration of probiotics and prebiotics, and fecal microbiota transplantation (FMT).

Dietary factors are pivotal with regards to the composition of human mycobiome and bacteriome, and the risk of specific types of cancer in the future. As vegetarians have been found to possess different compositions of their mycobiome, in comparison to subjects who consume a Western-based nutrition, it is highly likely that the nutritional status may represent a significant way of preventing these specific types of cancer [93].

The human mycobiome, just like the human microbiome, is greatly influenced by dietary factors [94,95,96]. Hoffman et al. gathered 98 samples from healthy participants to assess the association between the type of nutrition and the composition of intestinal microbiota [49]. Interestingly, the gut mycobiome was linked only to the recent type of nutrition. *Candida* was positively correlated with the consumption of carbohydrates, particularly polysaccharides and araboxylan, and was negatively associated with total saturated fatty acids, whilst *Aspergillus* was negatively related to the intake of short-chain fatty acids [49]. Intake of animal food was correlated with *Saccharomyces* [10].

In analogy to the favorable role of bacterial probiotics and prebiotics, fungal probiotics have shown beneficial anti-neoplastic effects, and could be helpful in cancer prevention and therapeutics [67]. The probiotic cocktail of *Saccharomyces*, *Lactobacillus rhamnosus*, *Lactobacillus acidophilus* and *Bifidobacterium breve* has been shown to exhibit anti-biofilm and anti-tumor actions in the colon [97]. Galinari et al. have shown the antioxidant and pro-apoptotic properties of the yeast *Kluyveromyces marxianus*, that presents a phylogenetic association with *Saccharomyces cerevisiae. S. cerevisiae* is generally employed in the classic nutrition industry [98]. In another study, the cytoprotective actions of β-glucan, originating from *S. cerevisiae,* played an important role in the prevention of genotoxicity [99]. Notably, the treatment of animal models with selenium-enriched *S. cerevisiae* has been shown to exhibit a remarkable efficiency in comparison to the separate administration of selenium or *S. cerevesiae*. [100].

In line with the properties of *S. cerevisiae*, the commonly employed probiotic *Saccharomyces boulardii* has been reported to arrest the expansion of bacteria by synthesizing elevated concentrations of acetic acid [101]. Chen et al. have demonstrated that *S. boulardii* regulated inflammatory responses and suppressed gut cancer expansion in a mice model, by inhibiting the EGFR-Mek-Erk signaling network, while it exhibited pro-apoptotic actions in tumor cells by suppressing Akt, a central actor of the cell cycle [102,103]. Besides, *S. boulardii* has been shown to sufficiently decrease levels of inflammation and rebuild gut microbiota, resulting in an amelioration of cancer-associated colitis [102,103]. Apparently, beneficial fungi exist and seem to be capable of creating a favorable environment for anti-cancer effects. To date, *Schizophyllum commune*, *Saccharomyces cerevesiae* and *Saccharomyces boullardii* have been mainly reported to exert beneficial and anti-oxidant properties. Moreover, treatment targeting immune components, such as MBL or the complement C3 cascade, may prove to be effective in the therapeutic armamentarium against cancer [102,103].

Finally, FMT, which is the process of transferring a fecal microbiome from a healthy donor to another subject, restoring gut microbial homeostasis, may be a promising tool in CRC prevention and treatment. The composition of stool mycobiota of the donor could represent an important parameter in the efficacy of FMT as a preventive and therapeutic approach in CRC, as it was shown in other diseases such as *Clostridium difficile*-associated diarrhea and ulcerative colitis [104,105]. Indeed, the efficacy of FMT in these disease entities was associated with an increased load of *Saccharomyces* and *Aspergillus*, and a lower load of *Candida* in donor feces [105]. FMT with donor stool enriched with beneficial fungi may be also used as a potential adjunct treatment in augmenting the efficacy of immunotherapy with anti-PD-1 agents in epithelial cancer, including CRC [67,106].

More large-scale and long-term randomized placebo-controlled trials are needed to examine the safety, efficacy and sustainability of results in all these preventive and therapeutic interventions. Based on detected fungal signatures, novel targeted treatment modalities in precision medicine may emerge with the alteration or restoration of a healthy fungal community in patients with cancer.

### 6.2. Fungal Dysbiosis and Biomarkers as a Diagnostic and Prognostic Tool in Cancer

Alterations in the intestinal mycobiome could be used as an adjunct screening, diagnostic and prognostic tool in CRC, distinguishing early from advanced stages. Fungal biomarkers and dysbiosis could differentiate cases with CRC from healthy subjects, as was shown in a study where an altered load of 14 fecal fungal biomarkers, including an augmentation in *Malasseziomycetes* and a reduction in *Saccharomycetes* and *Pneumocystidomycetes*, presented a good diagnostic discriminative ability in CRC diagnosis [43]. The ratio of *Basidiomycota* to *Ascomycota* was also increased [43]. A proteomic study examining the contribution of fecal microbial secretome in colorectal carcinogenesis has revealed the presence of unique proteins from *Schizosaccharomyces pombe* in subjects with CRC, where 4 fungal proteins characterized the advanced stage [107]. Interestingly, a number of fungi detected in the oral cavity of individuals suffering from CRC, such as *Rhodotorula*, were indicative of CRC progression based on their enzymatic activity [108].

However, it is too early to draw conclusions regarding the real clinical significance and relevance of fungal dysbiosis and fungal biomarkers in cancer. It is of paramount significance to investigate if fungal dysbiosis is a causal factor in cancer promotion and progression, or a mere consequence of cancer development. Based on detected fungal signatures, novel targeted treatment modalities in personalized medicine may emerge with the modification or restoration of a healthy fungal community in patients with cancer.

## 7. Conclusions

The human mycobiome from the oral cavity to the gut has been suggested to be implicated in carcinogenesis, based on the fact that some fungal species are in abundance in healthy subjects, in sharp contrast to subjects with HNCC, colorectal and pancreatic malignancies. Moreover, as is the case of HNSCC, there may be slight differences among the same species, i.e., different strains of the same species. Patients with HNSCC, CRC and PDA harbor different fungal species than normal subjects, while the very recent study by Aykut et al. regarding PDA is suggestive of the crucial role of the activation of MBL-C3 cascade in the etiopathogenesis of PDA [44]. The inter-kingdom interplay between the human bacteriome and the human mycobiome may unravel novel pathways which could explain many unanswered questions. In the meantime, multi-omics studies are mandatory in the difficult task to find potential biomarkers and therapeutic targets regarding cancer and the human mycobiome. As our knowledge regarding the plausible associations between the human mycobiome and cancer is still expanding, emerging information will shed light upon this intriguing issue. More large-scale, longer-term, prospective and longitudinal studies with a multi-omics approach are required to examine the role of the mycobiome in the etiopathogenesis of cancer, and to delineate whether changes that occur in the mycobiome are causal or a consequence of cancer.

## Figures and Tables

**Table 1:** List of main studies associating the mycobiome with various types of neoplasms in animal models and humans.

Research/Year	Population, Type of Study	Clinical Specimen	Main Findings	Remarks
Head and Neck Cancer				
Perera et al., 2017 [26]	52 individuals; 25 with OSCC; 27 intra-oral-fibro epithelial polyps	52 biopsies from 25 patients with OSCC and 27 with oral polyps. DNA was extracted and sequenced for the ITS2 region	364 species accounting for 160 genera and 2 phyla (*Ascomycota* and *Basidiomycota*) were detected. *Candida* and *Malassezia* made up 48% and 11% of the average fungal community, respectively, according to Luan et al., 2015.	-5 species and 4 genera were identified in more than half of samples. -Less abundance and diversity in OSCC tissues of patients. *-Candida*, *Hannaella*, and *Gibberella* were ↑↑ in OSCC; *Altenaria* and *Trametes* were in greater quantity in polyps specimens. -*Candida albicans*, *Candida etchellsii*, and *Hannaella luteola*–like species were enriched in OSCC *Hanseniaspora uvarum*–like species, *Malassezia restricta*, and *Aspergillus tamarii* are predominant in polyps specimens. -Dysbiotic mycobiome dominated by *C. albicans* has been observed in OSCC.
Mukherjee et al., 2017 [25]	39 participants with OSCC of the tongue	39 tissue samples from oral SCC and adjacent tissues were analyzed after DNA extraction for 16S/18S rRNA gene.	Fungal richness was ↓↓ in tumor tissue (TT) in comparison to the adjacent non-cancerous tissue (ANCT), *p* < 0.006. The presence of 22 bacterial and 7 fungal genera was different in TT and ANCT. *Aspergillus* in TT was negatively associated with the presence of bacteria *Actinomyces*, *Prevotella*, *Streptococcus*, whilst it presented a positive association with *Aggregatibacter*.	-Subjects with advanced T-stage disease presented reduced mean differences between TT and ANCT, in comparison to subjects with regional disease. -Findings indicative of differences in the bacteriome and mycobiome between OSCC patients and their adjacent non-cancerous oral epithelium -Association with T-stage. -Despite the similarities in the index of diversity of the mycobiome between TT and ANCT, the abundance of the mycobiome was diminished in TT. -This study is suggestive of existing changes in the local environment in patients with OSCC, expressed as specific bacterial and fungal dysbiosis
Vesty et al., 2018 [27]	30 participants, including 14 patients with HNSCC	Saliva specimens analyzed by 16S rRNA gene and ITS1amplicon sequencing	↑↑ *Candida* *Candida albicans* representing more than 96% of fungi in the majority of subjects with HNSCC.	-↑↑ IL-1β and IL-8 in HNSCC and patients with poor dental health, when compared to healthy controls. -IL-1β and IL-8 levels were associated with *C. albicans*. -In HNSCC, salivary microbial and inflammatory markers are affected by oral hygiene.
Shay et al., 2020 [24]	92 individuals, including 46 patients with HNSCC	Oral wash samples analyzed by 16S rRNA and ITS gene sequencing	Distinct strains of *Candida albicans* are increased or decreased in oral wash specimens from patients with HNSCC, when compared to healthy controls.	-Distinct strains of *Candida albicans* and *Rothia mucilaginosa* differed in numbers. *Schizophyllum* commune was decreased in HNSCC patients, in comparison to healthy controls. -Compared to controls, oral cavity of subjects with HNSCC presents distinct differences in the mycobiome and bacteriome, and their interactions.
Colorectal Cancer				
Luan et al., 2015 [40]	27 patients with colorectal adenomas	Biopsies from colorectal adenomas and adjacent tissues were studied by using denaturing gradient gel electrophoresis (DGGE)	↑↑ *Ascomycota*, *Glomeromycota* and *Basidiomycota*. ↓↓ diversity in adenomas compared to adjacent tissue	-↑↑ *Basidiomycota* in adjacent tissues. -↑↑ *Basidiomycota* and *Saccharomycetales* in advanced adenoma samples, when compared to non-advanced.
Gao et al., 2017 [41]	131 individuals with colorectal carcinoma (CRC), colorectal polyps and normal subjects	Stool samples from patients with CRC, polyps and normal subjects were analyzed by using ITS2 gene sequencing	↑ ↑ *Ascomycota* followed by *Basidiomycota* ↓↓ diversity in the polyp group, when compared to controls.	↑↑ Ratio of *Ascomycota* to *Basidiomycota* in subjects with CRC and polyps, in comparison to controls. ↑↑ of the opportunistic fungi *Trichosporon* and *Malassezia*, which could be implicated in the progression to CRC.
Richard et al., 2018 [42]	27 patients with CRC; 7 with colitis-associated cancer, 10 patients with sporadic cancer and 10 healthy individuals	Tissue specimens from colonic resections in colitis-associated malignancy and sporadic CRC groups were analyzed using 16S rRNA and ITS2 sequencing	↑↑ *Basidiomycota* followed by *Ascomycota* ↓ diversity in sporadic cancer.	↑↑ *Basidiomycota* in colitis-associated cancer.
Coker et al., 2019 [43]	585 individuals; 184 patients with CRC, 197 patients colorectal adenomas and 204 normal subjects	Stool samples from patients with CRC, colorectal adenomas and normal subjects were analyzed by fecal shotgun metagenomic sequencing	-*Ascomycota*, *Basidiomycota* and *Mucoromycota* in patients with CRC and healthy participants. -No difference in diversity	-↑↑ *Basidiomycota/Ascomycota* ratio in CRC when compared to controls. -14 fungi identified with differential composition between CRC and controls.
Pancreatic Cancer				
Aykut et al., 2019 [44]	(1) Experiments in mice as well as in humans using 18S rRNA sequencing KC mice, which develop spontaneous pancreatic cancer by targeted expression of mutant Kras. C57BL/6, MBL-null, and C3−/− mice. (2) Human stool samples and pancreatic tissue specimens were gathered from healthy volunteers and subjects undergoing surgery for PDA or benign pancreatic disorder.	Because of the direct proximity and relationship of the intestinal and pancreatic duct via the Oddi sphincter, gut fungi could enter the pancreas. To examine this hypothesis, they administered GFP-labeled *Saccharomyces cerevisiae* to controls or cancer-bearing mice through oral gavage. Fungi moved into the pancreas in less than thirty minutes, suggesting that the intestinal fungal community may directly impact on the pancreatic microenvironment.	-PDA tumors harbored a ~3000-fold augmentation in fungi, in comparison to physiologic pancreas in both mice and humans. -PDA mycobiome was different from gut or physiologic pancreatic mycobiome based on diversity indexes. -The fungal community infiltrating PDA was ↑↑ enriched in *Malassezia* in mice and humans. -Fungal elimination with the use of amphotericin B was tumor-protective in slowly progressive as well as in models of invasive PDA, whereas re-population with *Malassezia* but not *Candida*, *Saccharomyces*, or *Aspergillus*–promoted oncogenesis.	-Connection of mannose-binding lectin (MBL), that attaches fungal wall glycans to activate the complement pathway, was needed in the promotion of malignancy. -MBL or C3 deletion in the extra-tumoral area or C3aR knockdown in tumor cells prevented tumor expansion. Reprogramming of the fungal ecosystem did not change PDA progression in MBL or C3 deficient mice. -Pathogenic fungi may promote PDA by activating the complement pathway via MBL induction.

Abbreviations: CRC: Colorectal Carcinoma; DGGE: Denaturing Gradient Gel Electrophoresis; HNSCC: Head and Neck Squamous Cell Carcinoma; IL: interleukin; ITS: Internal Transcribed Spacer; KC mice: mice that express Kras oncogene in their progenitor pancreatic cells; MBL: Mannose-Binding Lectin; OSCC: Oral Squamous Cell Carcinoma; PDA: Pancreatic Duct Adenocarcinoma; rRNA: ribosomal Ribonucleic Acid.
